# Primary Retroperitoneal Teratoma in a Young Male: A Case Report

**DOI:** 10.7759/cureus.15376

**Published:** 2021-06-01

**Authors:** Charan Singh, Niladri M Raypattanaik, Ishan Sharma, Lileswar Kaman

**Affiliations:** 1 General Surgery, Postgraduate Institute of Medical Education and Research, Chandigarh, IND

**Keywords:** primary retroperitoneal teratoma, retroperitoneal germ cell tumour, benign teratoma, mature teratoma, dermoid teratoma

## Abstract

Primary retroperitoneal teratomas are rare non-seminomatous germ cell tumors that arise from embryonal tissues. They form only 5%-10% of all retroperitoneal tumors. These are usually asymptomatic or present as lump or mass with compressive symptoms. Most of the patients are diagnosed by characteristic computed tomography findings. The chances of malignant transformation are rare. Complete surgical resection is the definitive treatment for most patients.

We had a 19 years old young man, presented with pain abdomen and awareness of a lump in the right upper abdomen. Contrast-enhanced computed tomography (CECT) of the abdomen revealed a heterogeneous soft tissue mass in the retroperitoneum with calcification. He was successfully treated with en-bloc complete surgical resection. Histopathology confirmed benign mature teratoma including all three germ layers. The patient is doing fine at nine months of follow-up and planned for CECT abdomen.

Primary mature teratomas arise in the retroperitoneum due to failure of germ cells migration to their normal location. Germ cells undergo differentiation into various germ layers. Teratomas can be classified as mature, immature, or non-dermal based on their histopathological characteristics. Although complete surgical excision is the mainstay of treatment, malignant teratomas frequently recur. So, annual follow-up is recommended with imaging.

A classic mature teratoma requires careful examination and interpretation of the imaging. The amount of immature components determines outcome and recurrence in these patients so en-bloc surgical resection is the treatment of choice.

## Introduction

Teratomas are non-seminomatous germ cell neoplasms derived from embryonal tissue and have elements from two or more germ layers [1]. Although there is bimodal age of presentation, still most of the cases are seen in the first decade of life [1,2]. They are located either in the gonads or in the extra-gonadal sites like the sacrococcygeal region, anterior mediastinum, retroperitoneum, neck, and pineal gland [1,2]. Secondary from genitalia, soft tissue sarcoma, neurogenic mass, tuberculosis, Kaposi sarcoma, Castleman disease, and growing teratoma syndrome are the most commonly seen retroperitoneal tumors. Primary retroperitoneal teratomas (PRT) are very rare, representing 4% of all retroperitoneal tumors [1,3]. Most of the patients are diagnosed incidentally due to increased use of imaging for other reasons. Symptomatic patients present with pain abdomen and abdominal lump or mass [1]. Most of the mature teratomas are benign but malignant degeneration is not uncommon [1,2]. Large size and longer duration increase the chances of malignant transformation of mature teratoma [2]. Here, we are reporting a case of symptomatic primary retroperitoneal mature teratoma in a young man managed at a leading public sector-operated tertiary care center in northern India.

## Case presentation

A 19-year-old young man presented with a complaint of pain in the upper abdomen for two months. He also noticed a lump in the right upper quadrant during this period, which gradually increased in size. There was no significant past medical or surgical history. Family and personal history were unremarkable. He was moderately built and well-nourished with a BMI of 22.5 kg/m^2^. A 9 cm x 8 cm, ill-defined lump with firm consistency was palpated in the epigastrium and right hypochondrium on abdominal examination. Genitalia were normal on physical examination.

**Figure 1:**
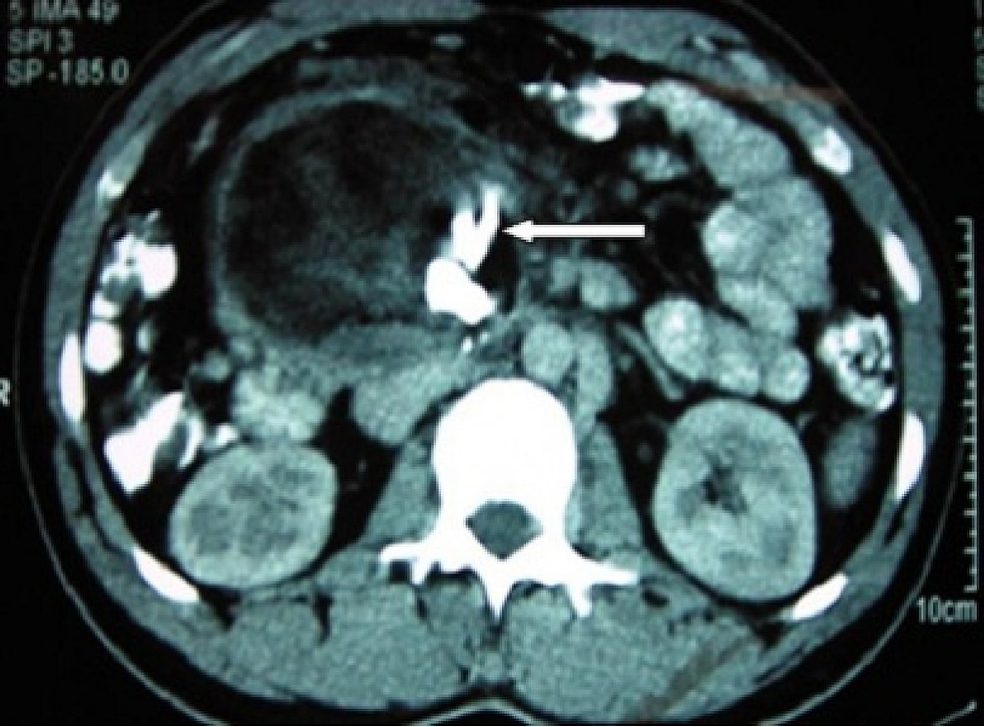
Contrast-enhanced computed tomography of the abdomen Contrast-enhanced computed tomography of the abdomen revealed an 8.6 cm x 7.7 cm heterogeneous soft tissue density mass from the retroperitoneum with calcification within (arrow).

Contrast-enhanced computed tomography (CECT) of the abdomen revealed an 8.6 cm x 7.7 cm heterogeneous soft tissue density mass arising from the retroperitoneum with calcification within, suggestive of teratoma (Figure 1). There was no retroperitoneal Lymph node enlargement. Tumor markers; lactate dehydrogenase (LDH), alpha-fetoprotein (AFP), and beta-human chorionic gonadotropin (beta-HCG) were within the normal range. All hematological and biochemical parameters were within the normal limits. Bilateral testicles were normal on ultrasound examination.

**Figure 2:**
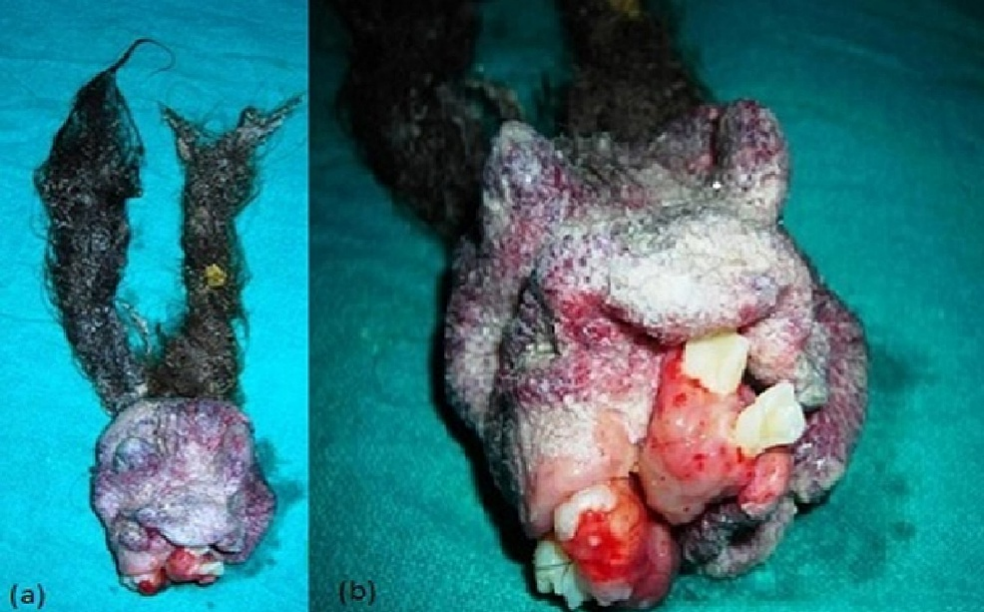
Post-excision specimen (a) and (b) A mass consisting of a long tuft of hair and putty materials, rudimentary eyes, ear, nose, jaws, and gum with fully developed teeth.

On surgical exploration, there was a well-encapsulated tumor in the retroperitoneum behind the duodenum and head of the pancreas, which was excised in en bloc. On grossing of the tumor, there was a mass consisting of rudimentary eyes, ear, nose, jaw, gum with fully developed teeth, a long tuft of hair, and putty materials (Figures 2a, 2b). Histopathology of the tumor was a benign mature teratoma with tissues derived from all three germ layers. The postoperative course was uneventful, and the patient was discharged on postoperative day 6. The patient is doing well on nine months of follow-up. Annual CECT abdomen is planned for a regular follow-up up to five years.

## Discussion

PRT are rare tumors with an incidence of 1%-11% [1]. Some studies have found that PRT accounts for 4% of all retroperitoneal neoplasms and is mainly seen in neonates and young adults [1,4]. They arise from germ cells that fail to migrate to normal locations. Germ cells are totipotent cells and they undergo differentiation into various germ layers [1,2]. PRT and metastasis from gonadal malignancy can be difficult to differentiate, but treatment and outcomes are different. So, exclusion of gonadal primary with clinical examination and imaging is a must before labeling a teratoma as PRT [3,5]. PRT usually presents as a single mass lesion, whereas metastasis from gonads can be multicentric as lymph nodal mass.

About 55% of patients with PRT present in the first decade of life [1,2]. The reported age of presentation varies from fetus to 82 years old with bimodal age of presentation with peaks in the first six months of life and early adulthood [1]. PRT is twice as common in females as in males [5]. The common site of presentation is in the retroperitoneum near the upper pole of the left kidney. They are generally asymptomatic, but they may present with abdominal pain, distension, nausea, vomiting, or compressive symptoms [5].

There is a risk of malignant transformation in 1% of PRT [2]. This risk is higher in adults than in children (26% vs. 10%) [3,6]. Malignant teratomas (0.2%-2%) have the potential to metastasize to the lung and lymph nodes [7]. Although there are no specific tumor markers for teratomas, some immature teratomas may have elevated serum AFP levels [8]. Diagnosis of PRT can often be made either by a CECT scan or by a magnetic resonance imaging scan. Calcification is a characteristic feature and is present in 74% of benign and 25% of malignant teratomas [5,6]. Imaging helps in not only diagnosing PRT but also delineating its relationship with adjacent structures, which is subsequently also useful in planning the surgery. Teratomas can be classified as mature, immature, or non-dermal [5,7]. Macroscopically cystic teratomas which contain mature tissue and sebaceous material are benign; while solid teratomas are composed of immature embryonic tissue along with fatty, cartilaginous, fibrous, and bony elements are frequently malignant [5,8]. Based on the excess of dermal elements in teratomas, they can also be classified as epidermoid (contain stratified squamous epithelium), dermoid (contain dermal elements like hair), or teratoid (contain columnar epithelium and sebum) [7]. The mainstay of the treatment is complete surgical resection. Despite complete surgical resection malignant teratomas frequently recur [5,8]. The reported five-year survival for benign teratomas is 100% and for malignant tumors, it is about 67% [7,9,10]. Differential diagnosis of various diseases likes a secondary from genitalia, soft tissue sarcoma, neurogenic mass, tuberculosis, Kaposi sarcoma, Castleman disease, and growing teratoma syndrome (development of a mature teratoma following chemotherapy for a non-seminomatous germ cell tumors) shall be considered [11]. Frequently, the diagnosis is based on imaging, but surgery and pathology are mainstays for the final diagnosis [11]. There is a risk of malignant transformation of teratomas, which may be the cause of relapse if a tumor is not excised completely during surgery. Late relapse is defined as recurrence after two years of completion of primary treatment [7]. These malignant variants respond poorly to adjuvant treatment. Patients should be followed up with an annual CECT or other abdominal imaging modalities (ultrasound or MRI) to identify recurrence at an early and asymptomatic stage, which can be treated by re-excision [7].

## Conclusions

PRT are rare tumors and usually diagnosed incidentally on imaging or symptoms due to mass effect, diagnosed by characteristic imaging appearance, and treated by en-bloc complete surgical resection. The amount of immature components determines the outcome and recurrence in these patients. Regular follow-up is recommended to detect early recurrence.
